# Impact of transit time on the reproductive capacity of *Euprymna scolopes* as a laboratory animal

**DOI:** 10.1186/s42826-022-00135-2

**Published:** 2022-07-30

**Authors:** Andrew G. Cecere, Tim I. Miyashiro

**Affiliations:** Department of Biochemistry and Molecular Biology, The Pennsylvania State University, University Park, PA USA

**Keywords:** Cephalopod, *Euprymna scolopes*, Squid, Symbiosis, *Vibrio fischeri*, Animal husbandry

## Abstract

**Background:**

The Hawaiian bobtail squid *Euprymna scolopes* hosts various marine bacterial symbionts, and these symbioses have served as models for the animal-microbe relationships that are important for host health. Within a light organ, *E. scolopes* harbors populations of the bacterium *Vibrio fischeri*, which produce low levels of bioluminescence that the squid uses for camouflage. The symbiosis is initially established after a juvenile squid hatches from its egg and acquires bacterial symbionts from the ambient marine environment. The relative ease with which a cohort of wild-caught *E. scolopes* can be maintained in a mariculture facility has facilitated over 3 decades of research involving juvenile squid. However, because *E. scolopes* is native to the Hawaiian archipelago, their transport from Hawaii to research facilities often represents a stress that has the potential to impact their physiology.

**Results:**

Here, we describe animal survival and reproductive capacity associated with a cohort of squid assembled from two shipments with markedly different transit times. We found that the lower juvenile squid counts generated by animals with the longer transit time were not due to the discrepancy in shipment but instead to fewer female squid that produced egg clutches at an elevated rate, which we term hyper-reproductivity. We find that hyper-reproductive females were responsible for 58% of the egg clutches laid.

**Conclusions:**

The significance of these findings for *E. scolopes* biology and husbandry is discussed, thereby providing a platform for future investigation and further development of this cephalopod as a valuable lab animal for microbiology research.

## Background

Bacteria have significant roles in promoting the normal development and physiology of animals [1]. The co-evolution of animals with bacteria have led to symbiotic interactions, *i.e.*, interactions that are highly specific and long-lasting. In fact, the human body establishes symbioses with many types of bacteria that contribute to fundamental processes, such as immunity and digestion, and perturbation to these symbioses often leads to states of disease [2, 3]. Increasing understanding of the mechanisms that contribute to the assembly, maintenance, and transmission of the bacterial symbionts of humans has the potential to combat disease and improve human health. However, the fact that so many of these bacterial symbionts function in the context of complex microbial consortia, *i.e.*, microbiomes, makes the elucidation of these mechanisms a formidable challenge. For decades, researchers have turned to nature for other examples of animal-microbe symbioses, which have become powerful models for revealing the basic principles underlying symbiosis [4, 5]. Consequently, efforts to document and improve the husbandry techniques associated with these animal hosts will accelerate discovery in the field of symbiosis, which in turn, will help inform strategies to target bacterial symbionts to improve animal and human health.

**Fig. 1:**
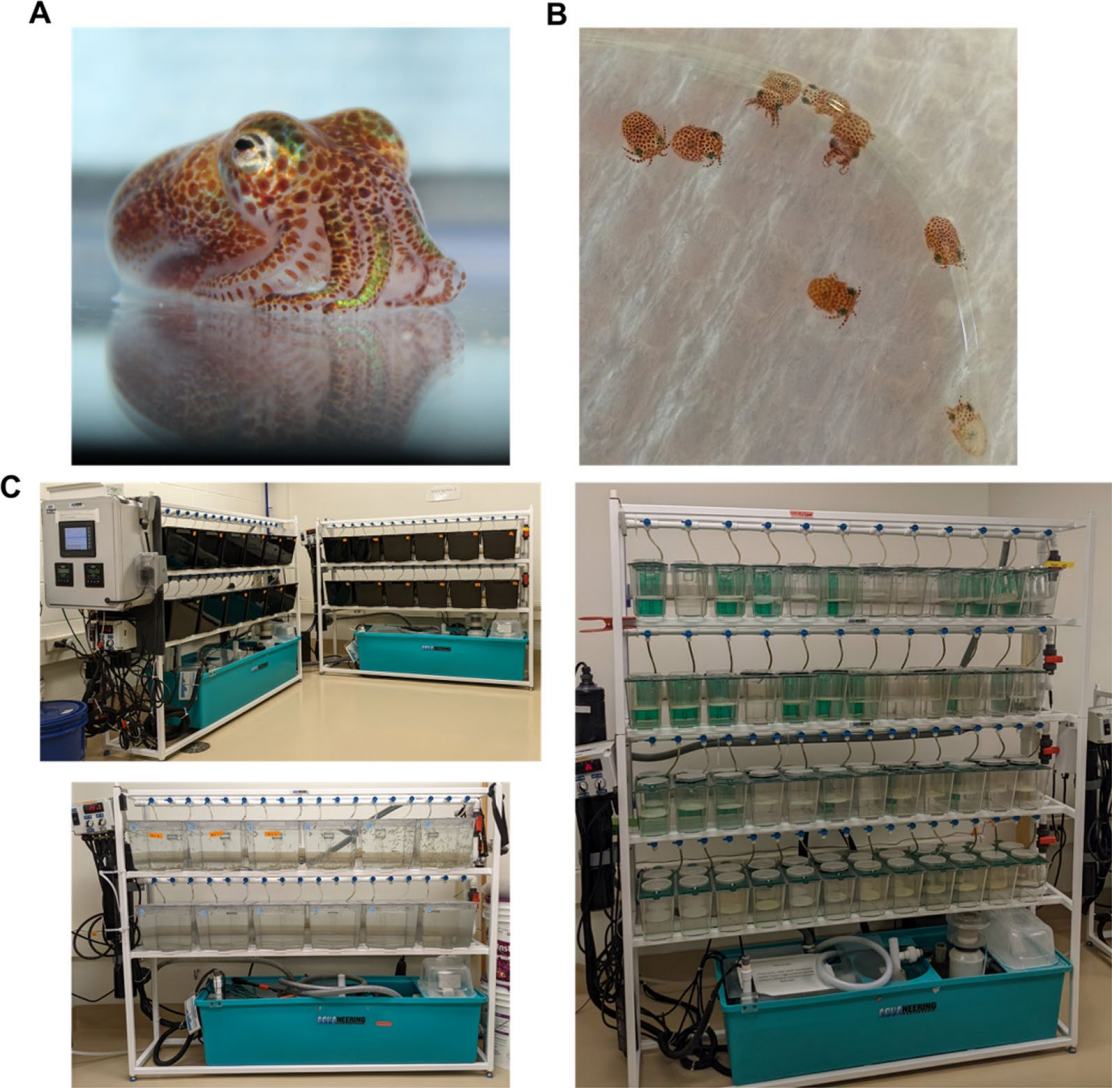
Mariculture facility associated with *E. scolopes* husbandry. **A** Adult *E. scolopes* squid. Typical mantle size is 25 mm. **B** Juvenile *E. scolopes* squid. Full length is typically 3–4 mm. **C** *Top left*, two 12-tank systems for housing adult squid. *Bottom left*, single 12-tank system for storing *P. paludosus* shrimp. *Right*, single 48 dual-chamber tank system for maintaining egg clutches

One well-studied example of an animal-microbe symbiosis is formed between the sepiolid (bobtail) squid *Euprymna scolopes* and the Gram-negative bacterium *Vibrio fischeri*. Adult *E. scolopes* (Fig. 1A) are endemic to the Hawaiian archipelago, where individuals are regularly observed at night in offshore water [6]. Populations of *V. fischeri* are housed within a light organ located inside the mantle cavity [7]. In exchange for host-derived nutrient and energy sources [8–10﻿], *V. fischeri* cells emit bioluminescence that the nocturnal host focuses downward as a camouflage technique to disrupt the shadow it casts within the water column [11]. Because this exchange is predicted to promote fitness of both partners, the interaction between the two taxa is a frequently cited example of a mutualistic symbiosis.

Over 30 years of research involving this symbiosis has led to *E. scolopes* becoming a powerful lab animal for investigating the molecular mechanisms by which bacteria establish symbiosis [12]. Juvenile *E. scolopes* squid hatch from their eggs without the bacterial symbiont (Fig. 1B), but they acquire environmental *V. fischeri* cells that colonize the light organ within a few hours [13]. Once the light-emitting symbiosis is established, the light organ undergoes morphological changes that prevent subsequent colonization events [14], and the symbiont populations resulting from these initial colonization events are maintained throughout the life history of the animal. Consequently, investigation into mechanisms by which the symbiosis is initially established depends on access to juvenile squid.

The demand for juvenile squid is typically satisfied by shipping a cohort of adult animals collected in Hawaii to the laboratory and maintaining them within a dedicated mariculture facility. Animals can be individually housed in standard aquaria (Fig. 1C), and upon combining male and female squid, mating occurs rapidly without pre-copulatory displays [15]. Females lay egg clutches that contain 12–300 eggs that are protected by a jelly coat stippled with an array of microbial symbionts with antifungal properties [15, 16]. Beginning at day 20, the mature embryos begin to hatch [17, 18]. Despite the increasingly frequent use of *E. scolopes* in microbiology research [12], significant knowledge gaps remain regarding how the husbandry associated with *E. scolopes* impacts the generation of juvenile squid. Furthermore, reports that describe studies on *E. scolopes* husbandry are rare, which prevents the working knowledge related to maintaining this organism in a laboratory setting from being disseminated to and discussed within the broader scientific community.

Establishing a cohort of wild-caught squid within a marine facility depends on the critical step of transporting them from Hawaii to the laboratory, which can be significant in distance. In this article, we report animal survival and reproductive capacity associated with a cohort of squid that was introduced into a mariculture facility following transit from Oahu, HI in two independent shipments. Because the transit times of the two shipments were markedly different, this cohort of animals provided the opportunity to assess the egg-laying productivity by female squid associated with each shipment group. We found that female squid associated with the delayed group laid fewer egg clutches overall, which resulted in the mariculture facility generating fewer juvenile squid. However, the difference in the number of egg clutches produced by each group was not due to the transit time but instead to the number of animals that exhibited a high frequency in laying egg clutches. Together, these findings reveal new insight into the biology and husbandry of *E. scolopes*, which will improve the use of this organism as a lab animal for microbiology research.

## Results

**Fig. 2:**
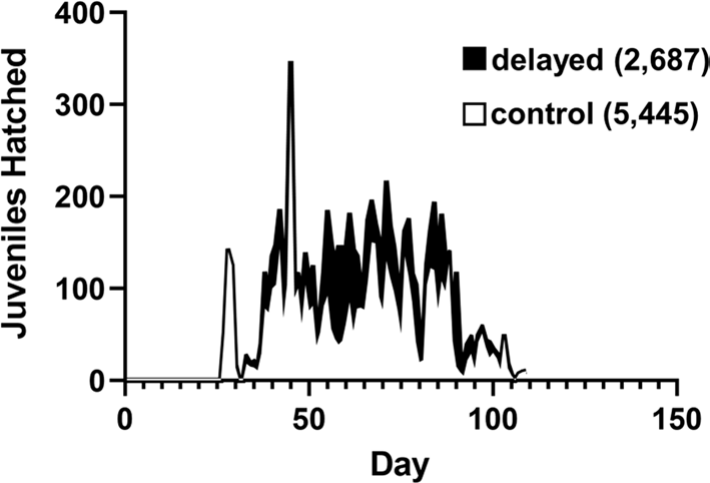
Total daily number of hatched juvenile squid associated with cohort. White (black) area indicates the daily number of juvenile squid that hatched from egg clutches associated with the control (delayed) group

A major purpose for *E. scolopes* as a lab animal is the generation of juvenile squid to serve as hosts for bacterial colonization experiments. Therefore, the number of juvenile squid generated by the facility can be used as a metric to assess the overall productivity of a particular cohort. This report focuses on a cohort of squid collected in August 2021, which featured 22 female and 7 male squid. Of note, the animals were shipped to the facility as two separate shipments: one shipment consisting of 11 females and 3 males that arrived in accordance with the planned transit time of 21 h (control) and the second shipment with the remaining 11 females and 4 males that had an atypical transit time of 45 h (delayed). Because the shipments arrived on the same day, all animals entered the mariculture facility on the same day. The water quality measurements associated with squid upon arrival were ammonia at 4–8 ppm, nitrite at 0 ppm, nitrate at 5 ppm, pH at 7.8, and salinity at 34–35 ppt, with no observable difference between shipments. With this cohort of adult squid, the mariculture facility generated a total of 8132 juvenile squid over a period of 80 days (Fig. 2). However, fewer juvenile squid hatched from clutches associated with the delayed group (2687 juvenile squid) than with the control group (5445 juvenile squid), which prompted further investigation into the dynamics of this cohort.

**Fig. 3:**
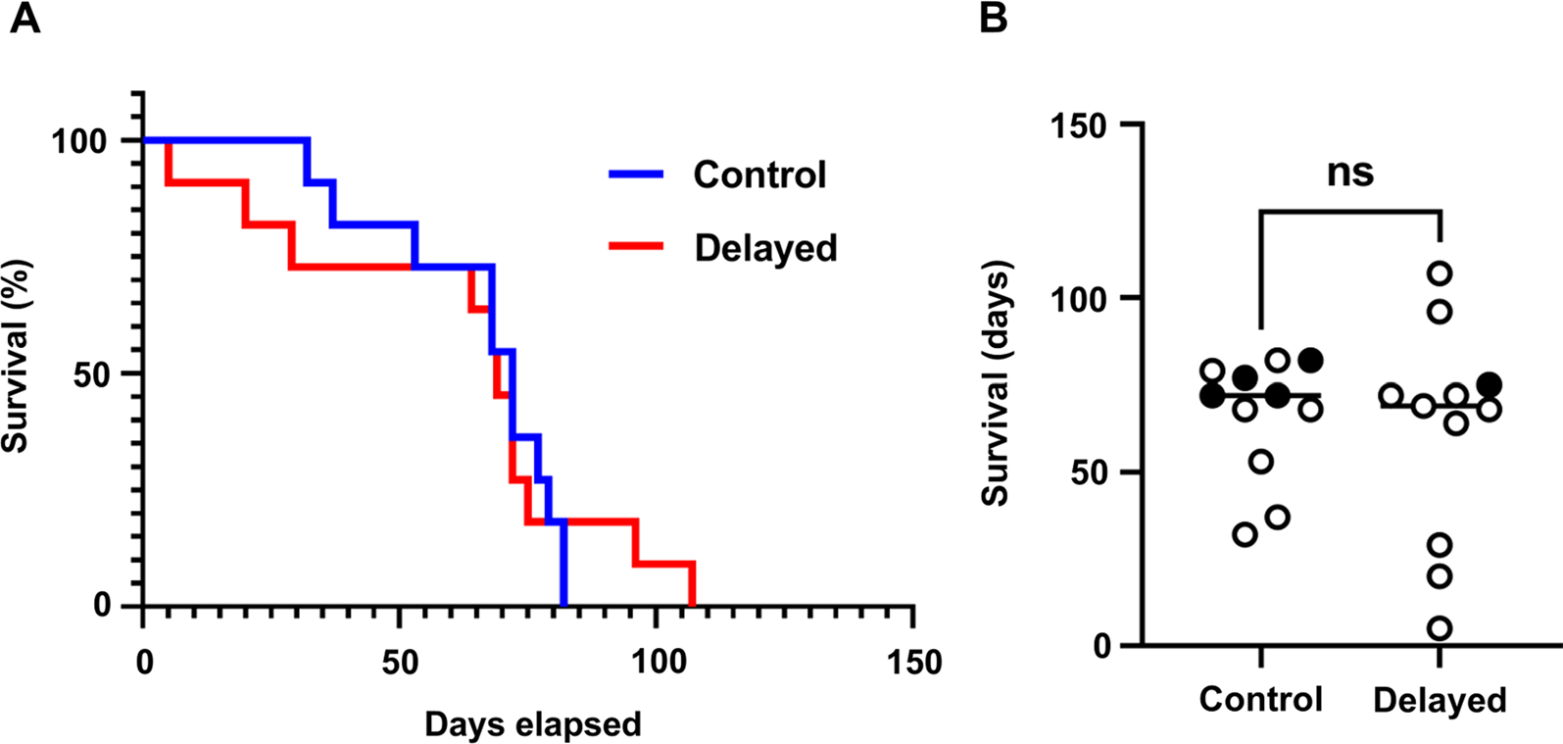
Survival of females from control and delayed groups. **A** Survival curves of control (blue) and delayed (red) groups (N = 11). Mantel-Cox test (chi^2^ = 0.004647, *df* = 1, *p* = 0.9457). **B** Each point represents the survival in days of one animal in the indicated group. Closed (open) symbols represent hyper-reproductive (non-hyper-reproductive) females. Unpaired, two-tailed Mann–Whitney test (N = 11/group, U = 53, n.s. = not significant with *p* = 0.6391)

**Fig. 4:**
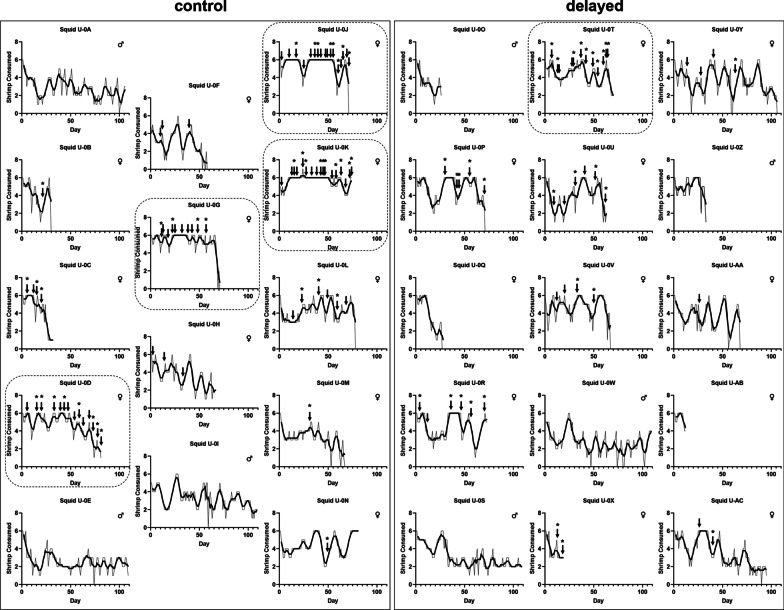
Feeding activity and egg clutch production by squid. Each graph displays the number of shrimp eaten daily by the indicated female squid [dark line = rolling average (N = 5 days)]. Arrows indicate when an egg clutch was laid, with the asterisks indicating egg clutches that were housed individually within a hatchery chamber (see Fig. 6). Graphs are organized according to control (left) and delayed (right) groups. Dashed outlines identify the hyper-reproductive females, i.e., produced at least 10 clutches

The cohort was maintained in the mariculture facility for 110 days. For the females, the median duration of survival was 70.5 days (Fig. 3A), and no difference in survival was detected between delayed and control groups (Fig. 3B). Crustaceans represent a primary dietary component of cephalopods [19], with *E. scolopes* squid preferring live shrimp as a food source. Using the commercially available freshwater shrimp *Palaemonetes paludosus*, which can survive in marine seawater for over 24 h, we maintained the cohort on a diet of *P. paludosus* and assessed individual feeding behaviour (Fig. 4). On average, squid associated with either shipment consumed 4 shrimp each day, with females eating approximately one shrimp more than males. No difference in shrimp consumption was observed between delayed and control groups (unpaired *t*-test, *p* = 0.9562). Taken together, these results suggest that the different transit times did not impact feeding behaviour or survival of the squid within the mariculture facility.

**Fig. 5:**
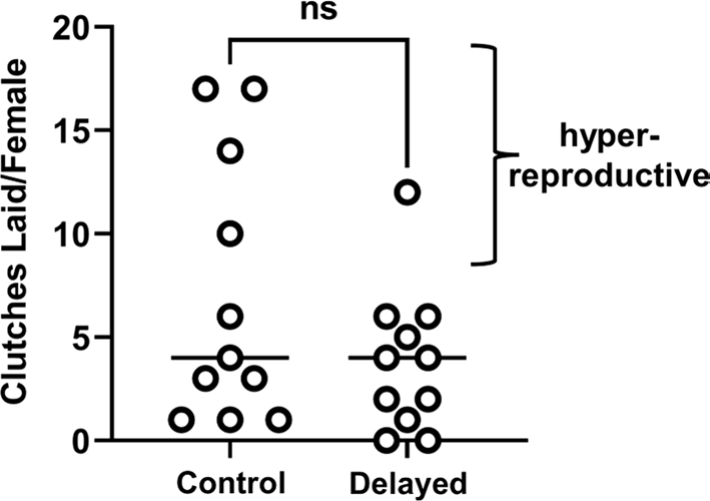
Clutches laid by female squid. Each point represents an individual animal (N = 11 per group). No statistical significance (ns) was detected between group medians (bars) based on a two-tailed Mann–Whitney test (U = 45.50, *p* = 0.3374)

**Fig. 6:**
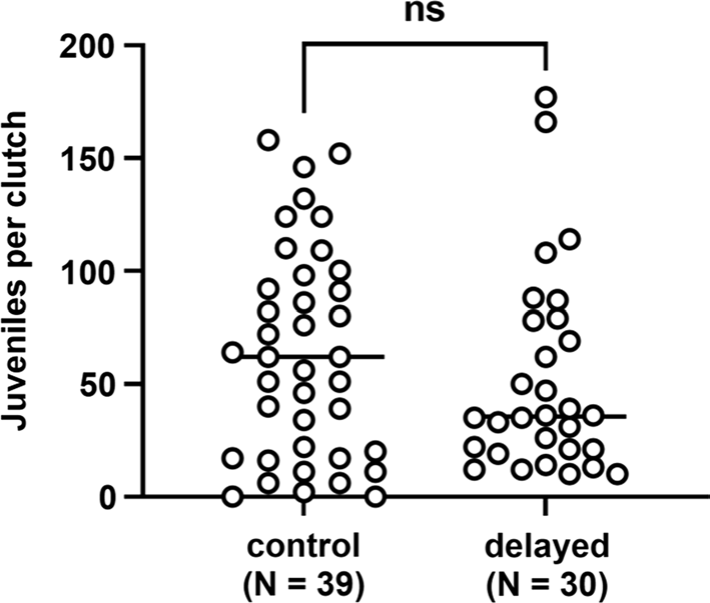
Juveniles generated from egg clutches. Each point represents an egg clutch. No statistical significance (ns) was detected between group medians (bars) based on a two-tailed Mann–Whitney test (U = 501, *p* = 0.3128)

Next, we sought to determine whether the transit time affected the generation of juvenile squid by each group. Over the course of 80 days, there were 119 egg clutches laid (Fig. 4, arrows). While the number of clutches laid by each female did not vary statistically between delayed and control groups (Fig. 5), there were fewer egg clutches laid by the delayed group (42 clutches) than the control group (77 clutches). We hypothesized that the discrepancy in egg clutches laid by each group could explain the corresponding difference in juveniles generated; however, another possibility was that transit time affected the number of juveniles produced by each clutch. Because 60 of the 119 total clutches were housed in hatchery chambers as pairs, only the 69 egg clutches that were housed individually could be used to address this other possibility. Of the 21 females that laid at least one egg clutch, 17 females (9 from the control group and 8 from the delayed group) had at least one egg clutch represented within the data set (Fig. 4, asterisks). The distribution of juveniles per egg clutch was not different between groups (Fig. 6), which suggests that the generation of juveniles by egg clutches was not affected by the different transit times. Taken together, these results suggest that the lower number of egg clutches laid by the delayed group was responsible for the fewer juveniles generated.

**Fig. 7:**
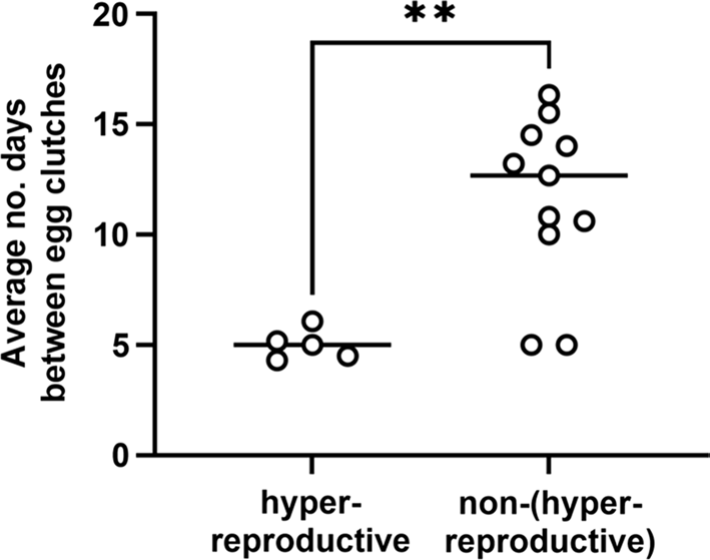
Egg clutch production rates. Points represent the average number of days between egg clutches for an individual animal. Statistical significance was detected between group averages (bars) based on a two-tailed Mann–Whitney test (U = 5, ** = *p* = 0.0087)

Closer inspection of the number of egg clutches laid by individual animals (Fig. 5) revealed that five females laid more than twice the median (4 egg clutches). In fact, 58% (70/119) of the clutches produced by the entire cohort were laid by those five animals alone. Because these animals (designated as hyper-reproductive squid) had a large impact on the overall production of egg clutches, we examined the properties of this group in more detail. On average, hyper-reproductive squid survived within the mariculture facility 74.4 ± 4.5 days (Fig. 3B) and laid an egg clutch every 5.0 ± 0.7 days (Fig. 7). The survival of non-hyper-reproductive female squid within the facility ranged from 13 to 110 days (median = 68.50 days) and on average exceeded 11 days between egg clutches (Fig. 7). Notably, two female squid initially laid clutches at a rate comparable to hyper-reproductive squid (Fig. 4, Squid U-0C and U-0X), but they only survived for 20 and 33 days, which prohibited them from contributing more than 2 and 4 clutches, respectively. Six female squid either laid a single clutch or failed to lay any clutches, which prevented calculation of a clutch-laying rate for those females. In addition, hyper-reproductive females ate an average of 5.0 ± 0.5 shrimp each day, which was greater than non-hyper-reproductive females (4.0 ± 0.6 shrimp) (two-tailed, unpaired *t*-test, *p* = 0.0030), which suggests a higher feeding intake associated with hyper-reproductive females. Taken together, hyper-reproductive squid featured a rapid rate of egg laying and an elevated rate of shrimp consumption.

**Fig. 8:**
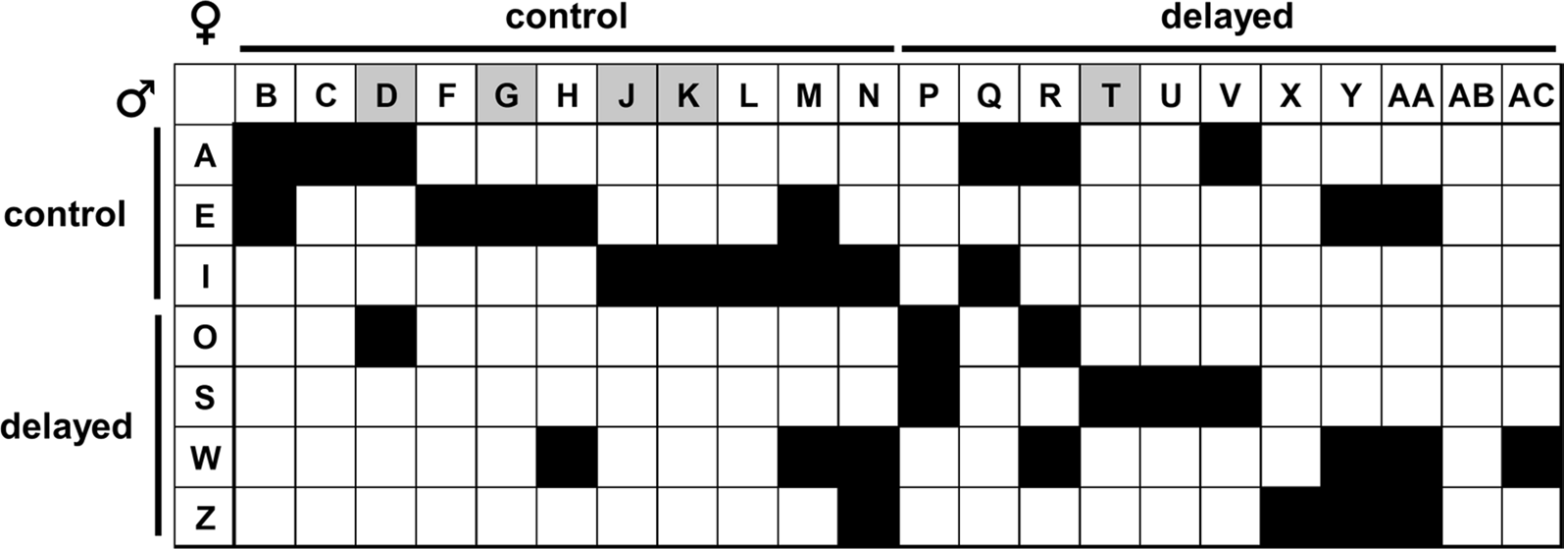
Mating pairs established in squid cohort. Each filled box indicates that at least one mating was observed between the corresponding male (row) and female (column). Animals are organized according to control and delayed groups. Hyper-reproductive females are indicated by gray boxes. Letters refer to suffixes of animal labels in Fig. 4

Establishing a productive cohort of *E. scolopes* within a mariculture facility typically includes the maintenance of several male squid for breeding purposes. Therefore, we considered whether the observed hyper-reproductivity was correlated with specific males. As described in the methodology, each female was mated with a male every 14 days. On average, males mated with 5.3 ± 1.6 females (Fig. 8), with 1.5 ± 1.1 pairings involving one individual from the control group and the other from the delayed group. Of the five males (A, E, I, O, and S) that had mated with hyper-reproductive females, only one (I) had mated with two different hyper-reproductive females (J and K). For each of these males, most females with which they mated were not scored as hyper-reproductive, which suggests that hyper-reproductivity was uncorrelated with specific males in the cohort. In addition, four of the hyper-reproductive females (G, J, K, and T) only mated with one male (E, I, I, and T, respectively), which suggests that the hyper-reproductivity is not due to a female being involved in pairings with different males.

Finally, to test whether the occurrence of hyper-reproductive females is reproducible, we examined previous cohorts for which data were available. For cohorts that were collected at least 18 months apart from one another, the frequency of hyper-reproductive females and their contribution to egg clutches were consistent at 23.1 ± 0.6% and 57.4 ± 2.8%, respectively (Table 1). Together, these observations suggest that occurrence of hyper-reproductive females associated with *E. scolopes* cohorts is remarkably consistent over time.

**Table 1:** Occurrence of hyper-reproductive females (HRF) among cohorts

Collection period (month, year)	No. HRF (total no. females)	No. HRF clutches/total clutches (percentage)
August, 2017	5 (21)	65/110 (59.1%)
March, 2019	5 (22)	65/120 (54.2%)
August, 2021†	5 (22)	70/119 (58.8%)

^†^Cohort examined in this study

## Discussion

This report describes the dynamics associated with a cohort of *E. scolopes* lab animals maintained within a mariculture facility for the purpose of generating juvenile squid for microbiology research. To our knowledge, there have been no reports in the scientific literature on whether the shipment of live *E. scolopes* to laboratories could impact their reproductive success in captivity. The extended transit time associated with one of the shipments of adult squid provided the opportunity to test whether the transportation phase of assembling a cohort impacts the generation of juvenile squid by the mariculture facility. No differences in survival or rate of laying egg clutches were observed between the two groups, which are important findings that are further discussed below. In addition, this study revealed that only a small subset of females was responsible for most egg clutches laid by the cohort, and the implications associated with the discovery of hyper-reproductive squid are considered in more detail.

The shipment process represents a major linchpin in assembling a cohort of squid within a mariculture facility due to the inherent reliance on third-party carriers to facilitate the shipment. Logistical disruptions have the potential to jeopardize cohort assembly by extending the transit time for specific shipments. Notably, the results reported here suggest that a transit time that extends up to 45 h does not interfere with the egg-laying productivity of the squid within a mariculture facility. Consequently, researchers likely have increased flexibility in planning shipments that take 21–45 h to complete.

An unexpected finding from our analysis of this cohort was that a subset of females had laid most of the egg clutches. This discovery of hyper-reproductive females is significant for multiple reasons. First, the increased rate of egg laying of hyper-reproductive females represents a new area of research in cephalopod reproduction to explore. In contrast to other members of the Sepiida order like cuttlefish [20], the factors that contribute to reproductive success of sepiolid squid are far less characterized. The recent assembly and annotation of the *E. scolopes* genome will likely serve as an invaluable resource when investigating whether a genetic basis for hyper-reproductivity exists [21]. Next, hyper-reproductivity has the potential to improve cephalopod usage in research, by lowering the number of females necessary to capture and maintain within a mariculture facility for the purpose of generating juvenile squid. Because data on the ecology of *E. scolopes* remains sparse, predictions of how the distribution of natural populations will respond to climate change cannot be accurately made. Consequently, increasing understanding of the traits associated with hyper-reproductive females, e.g., elevated feeding rates, may improve selection criterion that allows for increased stewardship of natural populations while promoting scientific advancement. Finally, the observation that certain females contribute more egg clutches within a mariculture facility has implications on research of the symbiosis with *V. fischeri*. Knowledge of which animals are hyper-reproductive enable the design of experiments that include pairing of different egg clutches to control for genetic variation between juveniles of different egg clutches [22]. Conversely, this knowledge can be implemented in other experimental designs by ensuring that experimental trials involve clutches laid by different females, thereby improving reproducibility in experimentation.

Previous work has demonstrated that juvenile animals can be reared to adulthood in a lab setting [14, 15], which raises the possibility of establishing a *E. scolopes* farm for research. However, such approaches require significant space and effort well beyond the mariculture facility described in this report. Therefore, further advancement of this research field depends on continued access to the juvenile squid generated by laboratory mariculture facilities. Consequently, the data reported here also establish a baseline for comparison with other animal cohorts, which will not only improve *E. scolopes* husbandry but will also better inform the protocols evaluated by institutional animal care and usage committees. Future studies will continue investigating various environmental factors associated with the mariculture facility and physiological parameters associated with wild-caught squid on the generation of juvenile squid, thereby facilitating scientific advancement with this powerful lab animal.

## Conclusions

This report describes the egg-laying productivity of *E. scolopes* individuals. Delayed shipment of wild-caught animals did not impact the rate of egg laying. A higher rate of egg clutch production was observed for a subset of animals, and this observation of hyper-reproductivity in *E. scolopes* has significance in improving animal husbandry and has revealed a novel area for future research.

## Methods

### Animal collection

Adult *E. scolopes* were collected using nets between August 12–18, 2021 at Maunalua Bay, Oahu, HI. Prior to shipment, animals were maintained at Kewalo Marine Laboratory in water tables containing natural sand and circulating Hawaiian sea water pumped from an offshore site (no known water quality issues at time of collection). Each water table contained a maximum of six squid per tank separated by sex. Animals were fed live ghost shrimp (*P. paludosus*) daily until the day of shipment.

### Animal shipment

Animals were shipped in double-bagged, 10″ × 22″ 3-mm aquarium shipping bags with 1.5 L seawater and the headspace replaced twice with oxygen. While most animals were bagged individually, smaller females were combined during the shipment process. Animals were shipped in climate-controlled cargo holds from Daniel K. Inouye International Airport (Honolulu, HI) to Pennsylvania State University (University Park, PA) in two secured, insulated crates via United Parcel Service (UPS) using the next-day air option on separate days. Crate #1 (11 females and 3 males) was shipped by filling out shipment information on-site and resulted in a total transit time of ~ 45 h (delayed group). Crate #2 (11 females and 3 males) was shipped using a pre-printed shipping label that resulted in no delay and total transit time of ~ 21 h (control group). Upon arrival, water chemistry of bags associated with three representative animals were assessed for each shipment. Measurements of ammonia, nitrites, nitrates, pH and salinity were taken. All squid from both shipments were alive and exhibited healthy behaviours (eating, burying, color).

### Acclimation

Prior to the arrival of the squid shipment, the water of the mariculture facility was assessed for temperature, salinity, ammonia, nitrite, nitrate, pH and adjusted as needed. Upon arrival, lights were dimmed in the mariculture facility room. The temperature of each bag was acclimated to that of the facility by floating the sealed bag within aquarium water. After 15 min, half of the animals began water acclimation by opening bags and dripping aquarium water at a rate of 33 mL/min into each bag. After 45 min, most of the water was removed from the shipping bag leaving the squid in ~ 500 mL mixed water. This process was completed once more before slowly inverting each bag and allowing squid to swim into individual aquarium tanks. After successful acclimation for half the animals from each shipment, this process was then completed for the remaining half.

### Mariculture system

Animals were maintained in a mariculture facility consisting of 4 separate aquarium systems designed and assembled by Aquaneering Inc. (2 adult systems, 1 egg system, and 1 shrimp system). Each system consists of modular polycarbonate tanks supported by a stainless-steel rack with a large filtration sump underneath. The sumps of both adult systems are interconnected, and each system contains approximately 60 gallons of mixed Instant Ocean seawater which circulates between the modular tanks, filtration sumps, and each system. Each adult tank contains on average one adult squid (marked with tape label), a 2-cm layer of Hawaiian sand, a 2″ PVC cave, and ~ 1.5 gallons of circulating seawater. The egg system contains approximately 60 gallons of Instant Ocean seawater circulating between 4 racks and 48 tanks. Each tank holds ~ 0.75 gallons of seawater split into 2 hatchery chambers with each chamber housing 1–2 clutches of eggs. As water from each system circulates through the sump below, it passes through a 3 GPM Self-Contained Filtration System comprised of the following: a fine aquarium filter media to catch large debris, a protein skimmer to remove organic waste, fluidized bed biofilter for nitrification, pelletized activated carbon for water purification, and a UV sterilizer to lower the microbial load. Water and equipment parameters such as water temperature (22 °C), pH (8.0–8.2), salinity (35 ppt), dissolved oxygen (8 ppm), water level and water leaks were continuously monitored via probes. In addition, ammonia, nitrite, and nitrate colorimetric test kits (API) were used to manually monitor water chemistry daily. Water changes were completed daily for both the adult and shrimp systems and bi-weekly for the egg system. Once per month, squid were housed outside of their tanks for approximately 1 h to allow for tank cleaning and the replacement of clean sand for each animal.

### Daily light cycle

Each day within the enclosed mariculture facility, lights fade on at midnight and off at noon to simulate the natural rising and setting of the sun in the natural environment. An additional dim static light is used to mimic moonlight.

### Feeding

Each adult squid was provided access to between 2 and 6 ghost shrimp daily based on their recent feeding behaviors. When a squid was found to have eaten all of their provided shrimp for two nights in a row, the number of shrimp given would increase by one the following day with a maximum of six shrimp. Alternatively, if any uneaten shrimp were found from the night before, the number of given shrimp would decrease by one the following day with a minimum of two shrimp. After ~ 18 h, any uneaten shrimp were identified to calculate the number eaten.

### Mating

Each mature female squid was mated with an individual male once every 14 days. To initiate mating, a male was transferred into a tank containing a female shortly after the lights had dimmed. Animals were periodically observed for mating and marked as ‘successful’ when mating occurred or marked as ‘unsuccessful’ if no mating was observed. After ~ 5 h, animals were separated by transferring the male back to their original tank. When possible, females were mated with a similarly sized male (or slightly smaller) from the same shipment. If a successful mating event did not result in egg clutch production, females were mated with a different male during their next mating period.

### Egg clutches/juveniles

Each day, PVC caves in the adult tanks were checked for newly laid egg clutches. When egg clutches were discovered, they were transferred to a separate egg system and removed from the PVC cave where they would incubate inside of hatchery chambers undisturbed. After 20 days of incubation, egg clutches were monitored twice daily for freshly hatched juvenile animals. Upon hatching, juvenile animals were transferred into filter-sterilized Instant Ocean seawater (FSSW) for experimental use.

### Data acquisition and analysis

Data were maintained and analyzed using Excel. GraphPad Prism v. 9.3.1 (GraphPad Software, LLC) was used to perform statistical analyses and generate graphs.

### Animal research protocol

Collection, care, and research of all laboratory animals was completed under the program’s Institutional Animal Care and Use Committee (IACUC). IACUC protocol # PROTO202101789.

## Data Availability

Data associated with this study are provided in the supplementary dataset file.

## Abbreviations

FSSW: Filter-sterilized Instant Ocean seawater
IACUC: Institutional Animal Care and Use Committee
